# IBUS‐SAS Is a Highly Accurate Intestinal Ultrasound Score for Predicting Endoscopic Disease Activity in Ulcerative Colitis

**DOI:** 10.1002/ueg2.70053

**Published:** 2025-05-29

**Authors:** Sarah Fischer, David Fischmann, Judith Wilde, Marcel Vetter, Laurin Wolf, Carol Geppert, Raja Atreya, Maximilian Waldner, Deike Strobel, Markus F. Neurath, Sophie Haberkamp, Sebastian Zundler

**Affiliations:** ^1^ Department of Medicine 1 University Hospital Erlangen Friedrich‐Alexander‐Universität Erlangen‐Nürnberg Erlangen Germany; ^2^ Deutsches Zentrum Immuntherapie University Hospital Erlangen Erlangen Germany; ^3^ Department of Pathology University Hospital Erlangen Friedrich‐Alexander‐Universität Erlangen‐Nürnberg Erlangen Germany

**Keywords:** bowel ultrasound, C‐reactive protein, endoscopic activity, fecal calprotectin, IBUS‐SAS, inflammatory bowel disease (IBD), limberg score, non‐invasive imaging, ulcerative colitis (UC), ultrasound score

## Abstract

**Background:**

The international bowel ultrasound group‐segmental activity score (IBUS‐SAS) is a validated tool with high interobserver agreement for accurately detecting disease activity in Crohn's disease (CD). Here, we addressed whether the IBUS‐SAS is also suitable to assess disease activity in ulcerative colitis (UC).

**Methods:**

The IBUS‐SAS and Limberg scores were determined in the sigmoid colon of patients with UC. The results were correlated to established scores of clinical, endoscopic and histologic disease activity (partial [pMS] and endoscopic [eMS] Mayo‐Score, ulcerative colitis endoscopic index of severity [UCEIS], histologic Nancy index) and/or biomarkers of inflammation (C‐reactive protein [CRP], fecal calprotectin). Sensitivity, specificity, positive (PPV) and negative predictive values (NPV) for IBUS‐SAS and the Limberg score to predict endoscopic and histologic disease activity were computed by receiver operating characteristics (ROC)‐analysis.

**Results:**

Fifty‐eight patients with UC were enrolled. The median IBUS‐SAS was 34.8. It was significantly correlated with pMS, eMS, UCEIS, Nancy index, CRP and fecal calprotectin. On ROC‐analysis, a cut‐off of 15.9 was reached with 100% sensitivity and 80.0% specificity for the prediction of endoscopic activity, resulting in a PPV of 94.7% and an NPV of 100%. The Limberg score performed only slightly worse (100.0%, 60.0%, 89.9%, 100%, respectively). Comparable results were found regarding the Nancy index for sensitivity (93.9% vs. 93.9%), specificity (57.1% vs. 42.9%), PPV (91.4% vs. 88.9%) and NPV (65.7% vs. 59.0%).

**Conclusions:**

This study highlights the potential of IUS for the non‐invasive quantification of disease activity in UC and suggests that the IBUS‐SAS should be considered as a diagnostic tool in trials and real‐world management of UC.

## Graphical abstract

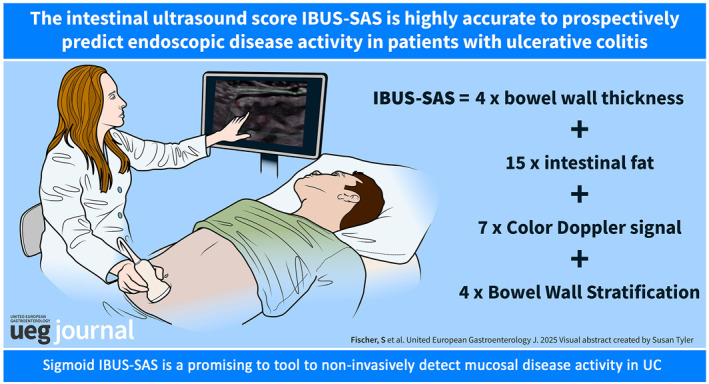

1

**Summary**

- Intestinal ultrasound is increasingly recognized for the quantification of disease activity in Crohn's disease (CD).
- There is a need for more data regarding intestinal ultrasound in ulcerative colitis (UC) patients.
- The international bowel ultrasound‐segmental activity score (IBUS‐SAS) was developed for the prediction of inflammation in CD with high inter‐observer accuracy and received external validation.
- This is the first study to investigate the accuracy of the IBUS‐SAS score to predict endoscopic and histologic disease activity in UC.
- This study demonstrates the value of the IBUS‐SAS as a diagnostic tool in UC.

## 2 Introduction

Inflammatory bowel diseases (IBD), primarily including Crohn's disease (CD) and ulcerative colitis (UC), are chronic conditions characterized by recurrent episodes of inflammation. For patients affected by these diseases, the impact on quality of life can be substantial and medical treatment or surgery is often required. To optimize the treatment for the individual patient, frequent objective assessments of inflammatory activity are essential. Following a treat‐to‐target regimen, it is common practice to periodically assess disease activity and adjust therapy accordingly, either by escalating (step‐up) or de‐escalating (top‐down) treatment, based on the results [1, 2].

Endoscopic follow‐up after initiating therapy remains the gold standard for monitoring inflammation, but the procedure's invasiveness, combined with the need for bowel preparation, often leads to reluctance among patients, especially those with a longstanding disease history [3]. Therefore, non‐invasive methods capable of accurately detecting disease activity are an important unmet need. Intestinal ultrasound (IUS) is widely available, low‐cost, reproducible, well tolerated by the patients and doesn't require extensive preparation [3, 4]. Transmural healing, a treatment goal associated with beneficial patient outcomes in CD, can be assessed using IUS and is defined as bowel wall thickness (BWT) of less than 3 mm in most studies [5, 6, 7]. Transmural healing correlates with reduced steroid use, fewer hospitalizations and lower need for surgery in CD [8]. Consequently, IUS‐response is increasingly being investigated in clinical studies [9]. To ensure high inter‐observer accuracy and reproducibility, IUS scoring systems must be developed and validated.

Multiple ultrasound features have been associated with inflammation in the gut. Historically, the Limberg score, which integrates BWT and colour Doppler signal (CDS), was one of the first scoring systems described [10]. Recently the international bowel ultrasound (IBUS) group identified the key sonographic parameters indicative of inflammation in CD, including BWT, bowel wall stratification (BWS), vascularization pattern using CDS and inflammatory mesenteric fat based on an international expert consensus [11].

**FIGURE 1:**
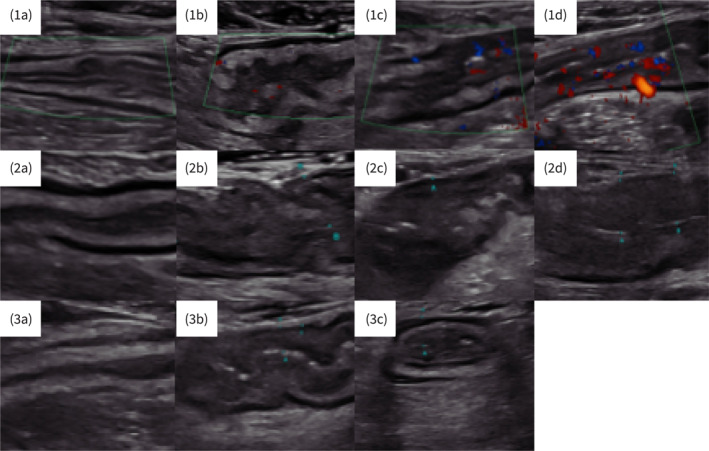
(1a–3c): Besides bowel wall thickness, the IBUS‐SAS consists of the components vascularisation, loss of bowel wall stratification (BWS) and mesenteric fat reaction. Regarding vascularization, the entering value can be either 0 (not increased, 1a), 1 (hypervascularization with a spotted pattern, 1b), 2 (hypervascularization with a streaky pattern, 1c) and 3 (hypervascularization involves the mesenteric fat, 1d). The parameter loss of BWS is categorized into normal (0; 2a), uncertain (1; 2b), focal (≤ 3 cm; 2; 2c), extensive (> 3 cm; 3; 2d) and mesenteric fat reactions in absent (0; 3a), uncertain (1; 3b) and present (2; 3c).

The IBUS‐segmental activity score (SAS) was then developed, utilizing a multivariable regression model to weight these parameters (Table 1, Figure 1). The parameter ‘loss of BWS’ is categorized into normal (0), uncertain (1), focal (≤ 3 cm; 2) and extensive (> 3 cm; 3), and ‘mesenteric fat reaction’ is categorized as absent (0), uncertain (1) and present (2). For the CDS, a modified Limberg score was adapted. The IBUS‐SAS follows the formula:

**TABLE 1:** Parameters of the IBUS‐SAS score (adapted from [11]).

Parameter	Graduation			
BWT	Mean of four two‐dimensional measurements			
i‐fat	0 = Absent	1 = Uncertain	2 = Present	
CDS	0 = Absent	1 = Short signals	2 = Long signals inside bowel	3 = Long signals inside and outside bowel
BWS	0 = Normal	1 = Uncertain	2 = Focal (</ = 3 cm)	3 = Extensive (> 3 cm)

IBUS‐SAS = 4 × BWT (mm) + 15 × intestinal fat + 7 × CDS + 4 × BWS [11].

The parameter BWT is the mean of four two‐dimensional measurements. IBUS‐SAS can theoretically range from 4 (no disease activity) to 100 (worst disease activity), with any computationally higher values being capped at 100. The IBUS‐SAS reached nearly perfect inter‐rater reliability, with an intraclass correlation coefficient of 0.97 (95% confidence interval [CI] 0.95–0.99), which was highly significant (*p* < 0.001) [11]. The authors recommended external validation of the score.

A recent study retrospectively analysed the diagnostic accuracy of IBUS‐SAS in 115 CD patients and found a strong correlation of IBUS‐SAS with clinical (Crohn's disease activity index [CDAI]), endoscopic (simple endoscopic score for Crohn's disease [SES‐CD]) and biomarker (faecal calprotectin [FC] and C‐reactive protein [CRP]) activity, with highly significant *p* values (< 0.001 for all categories). The IBUS‐SAS predicted endoscopic disease activity with a sensitivity of 73.7% and a specificity of 93.7% at a cut‐off value of 46.7 [12]. Similarly, another study demonstrated a positive correlation of the IBUS‐SAS and SES‐CD in 140 CD patients, with a sensitivity of 85.4% and specificity of 82.4% at a cut‐off value of 48.7, while a third study identified a cut‐off value of 25.2 with 82.2% sensitivity and 100% specificity [13, 14]. A strongly significant association (*p* < 0.01) with the CDAI and biomarkers was also observed [13].

However, there are conflicting data from a study that showed no association between IBUS‐SAS and the endoscopic picture. The retrospective study, which included 50 CD patients with ileal disease (60% with isolated ileal manifestation), found no correlation between IBUS‐SAS and endoscopic disease activity (SES‐CD), clinical (Harvey‐Bradshaw‐index [HBI]) or biochemical (fCP) activity [15]. The authors hypothesized that this lack of correlation was due to the ileal location and chronic thickening of the bowel wall despite the absence of inflammatory activity.

In ulcerative colitis other IUS scores were developed. In one approach, BWT and BWS and the presence of ulcerations graded from 1–4 for six colonic segments and the rectum determined disease severity in UC [16]. A multicentre study with 156 UC patients enrolled, showed significant correlations with clinical (*p* < 0.001), endoscopic (*p* < 0.001) and histologic (*p* < 0.001) findings for the score [16]. Likewise, the ulcerative colitis intestinal ultrasound index (UII), which includes BWT, CDS, bowel wall structure, loss of haustrations and mesenteric fat reaction for four bowel segments, was highly significantly associated with the endoscopic findings (*p* < 0.0001). The Milan ultrasound criteria (BWT, BWS, CDS, enlarged mesenteric lymph nodes, mesenteric fat reaction) discriminated active disease from remission in UC with a sensitivity of 85% and a specificity of 94% and these criteria received external validation [17].

Overall, IUS has been predominantly investigated in CD, likely due to the transmural involvement of inflammation. The above‐mentioned studies have clearly shown the potential of IUS in UC, with several IUS features associated with disease activity in CD being also accurate in UC and various established scores in either CD or UC. Still, there is an unmet need for a universally valid IUS score to assess disease activity.

Since it is derived from a systematic and broad international consensus, we hypothesized that the IBUS‐SAS is highly promising to provide such a harmonized evaluation of disease activity. Indeed, a potential role of the IBUS‐SAS in assessing disease activity in UC had already been explored in one cohort of UC patients and yielded promising results, although histologic data were not included [18, 19, 20]. Therefore, this study was designed to prospectively evaluate the accuracy of the IBUS‐SAS in UC patients, assess its ability to predict endoscopic and histologic disease activity, and compare it with the established Limberg score [10].

## 3 Methods and Materials

### 3.1 Study Design and Enrolment

This is a single‐centre, prospective cohort study investigating the correlation between the IBUS‐SAS and clinical, endoscopic, histological and biochemical disease activity in UC patients. Eligible participants were adults (≥ 18 years) with a confirmed UC diagnosis.

Patients were recruited from the outpatient IBD clinic, the endoscopy unit, and inpatient wards at the Department of Medicine 1, University Hospital Erlangen. All UC patients presenting there were invited to participate, regardless of clinical disease activity. Exclusion criteria included pregnancy, prior colectomy, ileostomy and an E1 phenotype (proctitis) (Table 2). Informed consent was obtained from all participants. The treatment regimen (drug, dosing and application interval) remained unchanged between IUS, endoscopy and blood sampling. All enrolled patients underwent IUS. The study procedures were approved by the ethics committee of the Friedrich‐Alexander‐Universität Erlangen‐Nürnberg (registration number 22–389‐B).

**TABLE 2:** Inclusion and exclusion criteria.

Inclusion criteria	Exclusion criteria
Established diagnosis of ulcerative colitis	Colectomy
Age ≥ 18 years	Ileostomy
Informed patient consent	Pregnancy
	Montreal classification E1 (proctitis)

### 3.2 Ultrasound Examination

All patients underwent IUS at the ultrasound unit of the Department of Medicine 1, University Hospital Erlangen, using a high‐frequency linear probe (Canon Aplio i11LX3) with a high‐sensitive preset. The Doppler preset was adjusted for low‐velocity flow and low pulse repetition frequency to maximize sensitivity, following the recommendations of the ‘European Federation of Societies for Ultrasound in Medicine and Biology’ (EFSUMB) [4]. The IBUS‐SAS components were measured in the sigmoid colon, recorded and the IBUS‐SAS was calculated. For BWT, the mean of four two‐dimensional measurements was used, including two longitudinal measurements spaced at least 1 cm apart and two cross‐sectional measurements taken at a minimum 90° angle between them, as recommended by the IBUS‐group [11].

The parameters CDS, intestinal fat and BWS were assessed individually by the observers. IUS investigations were conducted by a team of seven physicians (3 gastroenterologists, 4 internists) with extensive experience regarding IUS (> 5 years) in IBD patients. Investigators were blinded to endoscopic, histological and biochemical disease activity. IUS quality was also assessed and the date of the ultrasound examination along with clinical, endoscopic and biochemical markers were documented.

### 3.3 Clinical Disease Activity

Clinical disease activity was assessed using the partial Mayo score (pMS). A pMS of ≤ 1 indicated remission, whereas scores of 2–4 indicated mild, 5–7 moderate and values above 7 severe clinical activity [21].

### 3.4 Endoscopic and Histologic Disease Activity

Endoscopic disease activity was assessed using the endoscopic Mayo score (eMS) and the ulcerative colitis endoscopic index of severity (UCEIS), both of which are routinely evaluated in UC patients undergoing endoscopy in our department. Patients with an eMS of ≤ 1 were considered in remission, while a score of 2 indicated moderate and a score of 3 indicated severe endoscopic disease activity [22]. For the UCEIS, patients with a score of ≤ 1 were considered in remission, while scores of 2–4 were classified as mild, 5–6 as moderate and 7–8 as severe endoscopic disease activity [23]. Where available, the histological disease activity was recorded using the Nancy index. A Nancy index of ≤ 1 indicated histological remission, with all higher values indicating active disease [24].

### 3.5 Biomarkers of Inflammation

CRP and fCP were used as biomarkers of inflammation. A CRP level of ≤ 5 mg/L and a fCP ≤ 250 μg/g were considered normal, while higher values indicated active disease [25].

### 3.6 Statistical Analysis

All categorical variables (gender, Montreal classification, medical treatment, clinical, biochemical, endoscopic and histologic remission by category) are presented as counts and proportions. Metric variables are expressed as median and interquartile range (IQR).

The correlation between clinical, biochemical and endoscopic disease activity with the Limberg score and the IBUS‐SAS was analysed using Spearman's rank test. A *p* value < 0.05 was considered statistically significant. To visualize these correlations, scatterplots were created incorporating a linear regression line along with the 95% confidence interval (CI). Receiver operator analysis (ROC)‐analysis was employed to determine the sensitivity and specificity of the IBUS‐SAS in predicting endoscopic and histological disease activity and to identify optimal cut‐off values for IBUS‐SAS, including thresholds for moderate and severe endoscopic disease activity (as determined based on the UCEIS). Furthermore, the positive and negative predictive values for IBUS‐SAS and the Limberg score were calculated based on the sensitivity and specificity rates derived from the ROC analysis. All statistical analysis were conducted using GraphPad prism 10.

## 4 Results

### 4.1 Patient Characteristics

A total of 58 patients (35 female), with a median age of 31 (range 18–73), were enrolled.

Most patients had extensive and/or severe disease according to the Montreal classification (60.3% E3) and clinical, endoscopic and histological measures of disease activity, respectively (see Table 3 **for** all patient characteristics).

**TABLE 3:** Patient characteristics at the time of enrolment.

Patient characteristics				
Age [years]	31 (18–73)			
Gender	35 female (60.3%)			
	23 male (39.7%)			
Body‐mass‐index [kg/m^2^]	23.7 (16.6–55.9)			
Montreal‐classification	E1	*n* =	0	
	E2	*n* =	23	(39.7%)
	E3	*n* =	35	(60.3%)
Medication	None	*n* =	5	(8.6%)
	Mesalamine	*n* =	7	(12.1%)
	Steroids	*n* =	7	(12.1%)
	Azathioprine	*n* =	1	(1.7%)
	Infliximab	*n* =	14	(24.1%)
	Adalimumab	*n* =	2	(3.4%)
	Vedolizumab	*n* =	9	(15.5%)
	Ustekinumab	*n* =	3	(5.2%)
	Mirikuzumab	*n* =	3	(5.2%)
	Filgotinib	*n* =	2	(3.4%)
	Tofacitinib	*n* =	2	(3.4%)
	Upadacitinib	*n* =	3	(5.2%)
	Study medication	*n* =	2	(3.4%)

Note: The metric variables age and body‐mass index (BMI) are shown as median and range, while the categorical variables gender and medication are presented as counts (*n*) and proportions (%).

A majority (73.2%, *n* = 49) exhibited clinical disease activity, as reflected by a median pMS of 5 (IQR 1; 7) (Table 4). The biomarkers were elevated in 56.0% (*n* = 50) of the cases for CRP and 78.6% (*n* = 14) for fCP. The median CRP level was 5.8 mg/L (IQR 1.7; 18.5) and the median fCP was 743.8 μg/g (IQR 272.1; 1866.4). The time span between IUS and clinical data collection was a median of 0 days (IQR 0; 9), while for biomarker evaluation, it was a median of 1 day (IQR 0; 9).

**TABLE 4:** Clinical, biochemical, endoscopic and histological disease activity at enrolment.

Clinical, endoscopic and histological disease activity, biomarkers of inflammation				
Partial mayo score	Median 5 (IQR 1; 7)			
Clinical disease activity	Remission	*n* =	15	(26.8%)
	Mild activity	*n* =	12	(21.4%)
	Moderate activity	*n* =	21	(37.5%)
	Severe activity	*n* =	8	(14.3%)
Endoscopic mayo score	Median 3 (IQR 1; 3)			
Endoscopic disease activity (eMS)	0	*n* =	5	(11.1%)
	1	*n* =	7	(15.6%)
	2	*n* =	13	(28.9%)
	3	*n* =	20	(44.4%)
Ulcerative colitis endoscopic index of severity (UCEIS)	5 (IQR 3; 6)			
Endoscopic disease activity (UCEIS)	Remission	*n* =	10	(22.2%)
	Mild activity	*n* =	12	(26.7%)
	Moderate activity	*n* =	14	(31.1%)
	Severe activity	*n* =	9	(20.0%)
Nancy index	Median 3 (IQR 2; 4)			
Nancy score (category)	0	*n* =	5	(12.5%)
	1	*n* =	2	(5.0%)
	2	*n* =	5	(12.5%)
	3	*n* =	13	(32.5%)
	4	*n* =	15	(37.5%)
Faecal calprotectin [μg/g]	Median 743.8 (IQR 272.1; 1866.4)			
Biomarker activity (fCP)	Remission	*n* =	3	(21.4%)
	Activity	*n* =	11	(78.6%)
C‐reactive protein [mg/L]	Median 5.8 (IQR 1.7; 18.5)			
Biomarker activity (CRP)	Remission	*n* =	22	(44.0%)
	Activity	*n* =	28	(56.0%)

Note: Metric variables are shown as median and range, while categorical variables are presented as counts (n) and proportions (%). Clinical remission is defined as pMS of ≤ 1, while a pMS 2–4 is considered to be mild, a pMS of 5–7 moderate and higher values severe clinical activity [22]. Endoscopic remission is defined as UCEIS ≤ 1, while an UCEIS of 2–4 was considered to be mild, 5–6 moderate and 7–8 severe endoscopic disease activity [24].

Endoscopic disease activity was detected in 73.3% (*n* = 45) of the patients according to the eMS, while 77.8% had active endoscopic disease according to the UCEIS (Table 4). The median eMS was 2 (IQR 1; 3) and the median UCEIS was 5 (IQR 3; 6). The proportion of patients in remission with mild, moderate or severe endoscopic disease activity as categorized by the UCEIS was well‐balanced (Table 4). In most patients, IUS and colonoscopy were performed on the same day (median 0 days; IQR 0; 4). The Nancy‐index showed a median of 3 (IQR 2; 4), indicating that 82.5% of patients with available data had histological disease activity.

### 4.2 Correlation of the IBUS‐SAS With Individual Disease Activity Parameters

The median IBUS‐SAS was 34.8 (IQR 20.1; 59.5). The individual component BWT showed a median of 4.7 mm (IQR 3.7; 5.7). The respective proportions of each ultrasound parameter are summarized in Table 5.

**TABLE 5:** All IBUS‐SAS features are listed in this table.

IBUS‐SAS features				
Bowel wall thickness (BWT) [mm]	Median 4.7 (IQR 3.7; 5.7)			
Colour Doppler signal (CDS)	0	*n* =	27	(46.6%)
	1	*n* =	19	(32.8%)
	2	*n* =	10	(17.2%)
	3	*n* =	2	(3.4%)
Intestinal fat (i‐fat)	0	*n* =	30	(52.6%)
	1	*n* =	6	(10.5%)
	2	*n* =	21	(36.8%)
Bowel wall stratification (BWS)	0	*n* =	40	(70.2%)
	1	*n* =	10	(17.5%)
	2	*n* =	2	(3.5%)
	3	*n* =	5	(8.8%)
IBUS‐SAS	Median 34.8 (IQR 20.1; 58.2)			

Note: The IBUS‐SAS was calculated using the parameters bowel wall thickness (BWT), colour Doppler signal (CDS), inflammatory mesenteric fat (i‐fat) and bowel wall stratification (BWS) [11]. The CDS is categorized into no (0), short (1) or long Doppler signals within the bowel wall (2) or within and outside the bowel wall (3). I‐fat can be quantified as absent (0), uncertain (1) and present (2). The BWS is categorized as normal (0), uncertain (1), disrupted ≤ 3 cm (2) or > 3 cm (3) [11].

**FIGURE 2:**
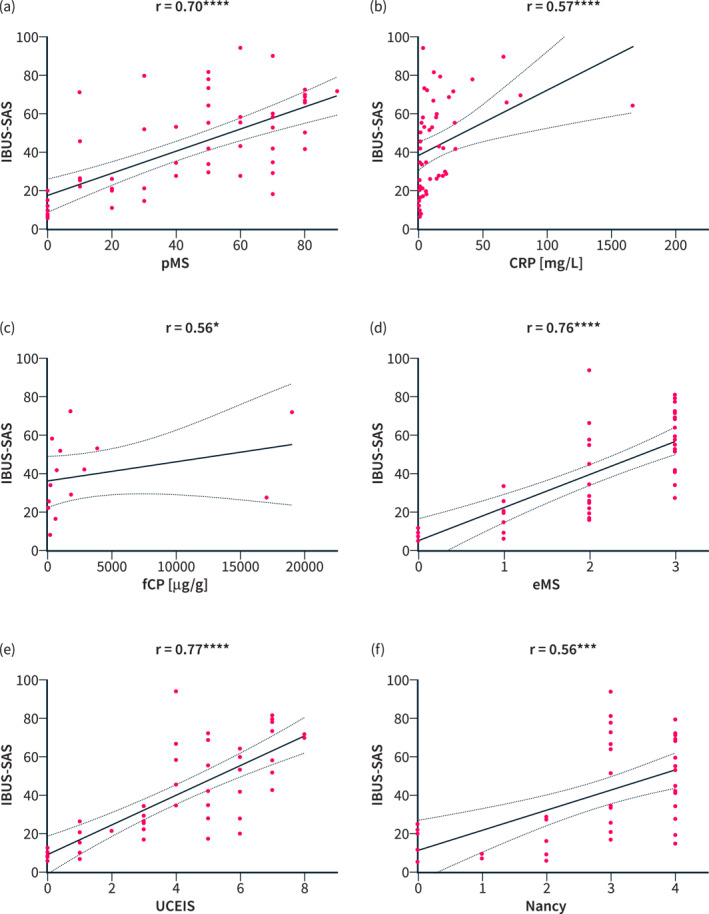
(a–f): Correlation of the IBUS‐SAS with clinical disease activity (a), the biomarkers CRP (b) and fCP (c) as well as the endoscopic (d: eMS, e: UCEIS) and histological inflammatory activity (f). Linear regression lines and 95% CI were added for illustration.

The IBUS‐SAS significantly correlated with each individual established readout of disease activity (Figure 2). Clinical disease activity, quantified with the pMS, showed a highly significant correlation with the IBUS‐SAS (*p* < 0.0001; *n* = 56; *r* = 0.70; 95% CI 0.52–0.81). Endoscopic disease activity was also highly significantly associated with the IBUS‐SAS with *p* < 0.0001 (*n* = 45; *r* = 0.76; 95% CI 0.59–0.86) for the eMS and (*n* = 45; *r* = 0.77; 95% CI 0.61–0.86) for the UCEIS. Similarly, the Nancy‐index showed a statistically significant positive correlation with the IBUS‐SAS with *p* = 0.0002 (*n* = 40; *r* = 0.56; 95% CI 0.30–0.75). The biomarkers were also significantly correlated with the IBUS‐SAS with *p* < 0.0001 (*n* = 50; *r* = 0.57; 95% CI 0.34–0.74) for CRP and *p* = 0.04 (*n* = 14; *r* = 0.56; 95% CI 0.19–0.84) for fCP.

### 4.3 Correlation of the Limberg Score With the Individual Disease Activity Parameters

For comparative purposes, the same correlations were analysed for the Limberg score. The Limberg score, likewise, was highly significantly correlated with clinical (*p* ≤ 0.0001; *n* = 56; *r* = 0.53; 95% CI 0.31–0.70) and endoscopic disease activity (*p* < 0.0001; *n* = 45; *r* = 0.70; 95% CI 0.50–0.82 for eMS and *p* < 0.0001; *n* = 45; *r* = 0.72; 95% CI 0.53–0.84 for UCEIS). The same was true for the biomarkers CRP (*p* < 0.0001; *n* = 50; *r* = 0.53; 95% CI 0.29–0.71) and fCP (*p* = 0.03; *n* = 14; *r* = 0.58; 95% CI 0.05–0.85). Similarly, the histological Nancy index was significantly correlated with the Limberg score (*p* = 0.003; *n* = 40; *r* = 0.46; 95% CI 0.17–0.68).

### 4.4 Comparison of Accuracy of IBUS‐SAS and Limberg‐Score for Predicting Endoscopic and Histological Disease Activity

Overall, the IBUS‐SAS demonstrated stronger correlations with most disease activity parameters compared to the Limberg score, particularly regarding clinical disease activity (*r* = 0.70 vs. 0.53), endoscopic findings (*r* = 0.76 vs. 0.70 for eMS; *r* = 0.77 vs. 0.72 for UCEIS), histological activity (*r* = 0.56 vs. 0.46) and CRP (*r* = 0.57 vs. 0.53). However, for fCP, the Limberg score showed a slightly stronger correlation (*r* = 0.56 vs. 0.58).

**FIGURE 3:**
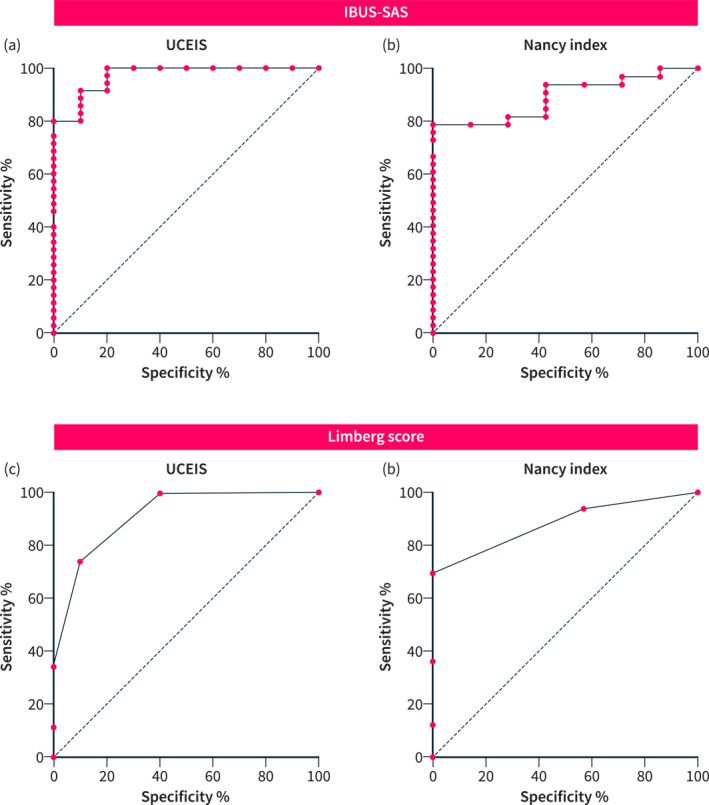
(a–d): ROC‐analysis of the IBUS‐SAS (a, b) and the Limberg score (c, d) regarding sensitivity and specificity for the prediction of endoscopic (a, c) and histologic (b, d) disease activity.

To determine whether the IBUS‐SAS and the Limberg score differ in their accuracy in predicting objective gold‐standard readouts, such as endoscopic or histological disease activity, ROC analysis was performed. The IBUS‐SAS showed high accuracy in predicting any endoscopic disease activity (100% sensitivity, 80.0% specificity, AUC 0.97) for a cut‐off value of 15.9 (Figure 3A). This resulted in a PPV of 94.7% and a negative predictive value (NPV) of 100%. Alternatively, a cut‐off of 20.9 was associated with 91.4% sensitivity, 90.0% specificity, 97.0% PPV and 75.0% NPV. In comparison, the Limberg score reached 100% sensitivity and 60.0% specificity (AUC 0.92) for a cut‐off of 0.5 (Figure 3C), resulting in a PPV for endoscopic activity of 89.9% and an NPV of 100%.

For histological activity prediction, the IBUS‐SAS reached a sensitivity of 93.9% and a specificity of 57.1% (AUC 0.89) for a cut‐off value of 13.7 (Figure 3B), yielding a PPV of 91.4% and an NPV of 65.7%. The Limberg score reached a sensitivity of 93.9% and specificity of 42.9% (AUC 0.88) for a cut‐off value of 0.5 (Figure 3D), leading to a PPV of 88.9% and an NPV of 59.0%.

### 4.5 The IBUS‐SAS for the Prediction of Endoscopic Disease Severity

We further examined whether the IBUS‐SAS can differentiate between mild, moderate and severe disease activity (Table 6).

**TABLE 6:** Proposed IBUS‐SAS cut‐off values for the discrimination of endoscopic disease activity with the respective sensitivities, specificities, PPV and NPV.

Endoscopic disease activity	Mild	Moderate	Severe
IBUS‐SAS cut‐off value	15.9	38.4	69.2
Sensitivity	100%	78.3%	66.7%
Specificity	80.0%	81.8%	94.4%
Positive predictive value	94.7%	81.7%	74.9%
Negative predictive value	100%	78.4%	91.9%

The IBUS‐SAS predicted moderate disease activity (78.3% sensitivity, 81.8% specificity, AUC 0.83) with a cut‐off value of 38.4, resulting in a PPV of 81.7% and an NPV of 78.4%. Severe endoscopic disease activity was predicted by the IBUS‐SAS (66.7% sensitivity, 94.4% specificity, AUC 0.89) with a cut‐off of 69.2, yielding a PPV of 74.9% and an NPV of 91.9%.

## 5 Discussion

The role of IUS in IBD is increasingly recognized, and while numerous studies have investigated its use in patients with CD, data regarding its application in UC remain underrepresented [3, 9, 10, 18, 26]. Since inflammation in UC is limited to the mucosa and submucosa, one might speculate that the ability to assess disease activity and response to therapy via IUS might be limited in UC compared with CD. However, prior studies have demonstrated that IUS parameters are also indicative of mucosal inflammation in UC and scores to assess disease activity in UC have been developed and partially validated [16, 17, 18]. Moreover, we hypothesize that the continuous pattern of inflammation in UC provides an opportunity for a highly standardized assessment of the sigmoid colon as a surrogate for overall disease activity. This in turn, may allow for a particularly precise and granular scoring system.

Response to therapy, as characterized by IUS, is increasingly recognized as an endpoint in clinical trials. However, to achieve consistent and comparable results, it is crucial to establish scores with high inter‐observer accuracy, sensitivity and specificity. Although several IUS scores to assess disease activity in UC and CD have previously been developed, the field is currently missing a harmonized and broadly applicable scoring system that can be used in both disease entities and might therefore simplify IUS training, provide consistent read‐outs across centres and settings and thereby also facilitate more multi‐centre trials.

The IBUS‐SAS was developed based on an international expert consensus for CD and external validation in CD as well as preliminary data in UC showed promising results [11, 12, 13, 18, 20]. Thus, our aim was to further evaluate the potential of the IBUS‐SAS to detect and score disease activity in UC. Indeed, our data demonstrate clear and significant correlations of the IBUS‐SAS with clinical, endoscopic, histological, and biochemical disease markers. This finding is consistent with most of the previously published results regarding the IBUS‐SAS [12, 13, 14, 20].

The optimal cut‐off identified in our study for distinguishing active versus inactive disease (as determined by the gold‐standard colonoscopy) was lower than in similar investigations of CD (15.9 vs. 25.2, 46.7 and 48.7), though the comparability is limited, since different endoscopic scoring systems are applicable [12, 13, 14]. Notably, using the cut‐off of 15.9, the IBUS‐SAS demonstrated a higher sensitivity compared to previous CD studies (100% vs. 73.7%, 85.4% and 82.2%), albeit with a slightly lower specificity (80% vs. 93.7%, 82.4% and 100%) [12, 13, 14]. While the alternative cut‐off of 20.9 showed a still high sensitivity (91.4%) and comparable specificity (90%), we believe that prioritizing a cut‐off with near‐perfect sensitivity is clinically more relevant. Consequently, an IBUS‐SAS below 15.9 effectively ruled out endoscopic disease activity in our cohort, underscoring the potential of IUS to non‐invasively exclude relevant disease activity and potentially obviating the need for invasive procedures in patients with IBUS‐SAS remission. Regarding the graduation of endoscopic disease severity, we propose additional cut‐off values of the IBUS‐SAS for moderate (38.2) and severe (69.2) endoscopic disease activity. However, their accuracy is somewhat lower compared to the discrimination between absent and present endoscopic disease activity.

As expected, the Limberg score also showed a significant correlation with all disease activity markers. However, IBUS‐SAS generally outperformed the Limberg score in terms of sensitivity and specificity for endoscopic (100% and 80% vs. 100% and 60.0%) and histological (93.9% and 57.1% vs. 93.9% and 42.9%) disease activity. Consequently, the IBUS‐SAS demonstrated slightly better performance in terms of PPV (94.7% and 91.4% vs. 89.9% and 88.9%) and NPV (100% and 65.7% vs. 100% and 59.0%) for endoscopic and histological activity, respectively. The fact that the Limberg score cut‐off was set at 0.5, and a score of 1 is reached only with increased bowel wall thickness, highlights the central importance of this parameter in detecting relevant disease activity. This observation aligns with findings during the IBUS‐SAS development [11]. Similar to the distribution of endoscopic disease activity, only a few patients exhibited normal BWT, while the majority were classified ‘negative’ or ‘uncertain’ for the parameters inflammatory mesenteric and BWS. Nevertheless, our data demonstrate that inclusion of additional parameters in the IBUS‐SAS substantially enhances the specificity and also PPV for predicting endoscopic and histological disease activity.

An important limitation of our study is the single‐centre design, reporting data from a tertiary care setting with extensive IUS experience. Therefore, it remains unclear whether these findings can be broadly reproduced, and further studies are necessary to address this. Another limitation is the relatively small sample size for fCP (*n* = 14), necessitating cautious interpretation of the correlation. Key strengths of our study include its prospective and well‐controlled design, systematic and highly standardized IUS investigations in patients with UC, and the comprehensive data set on all established disease activity markers, particularly clinical and endoscopic features, as well as the inclusion of histological data.

Although numerous IUS scores have been validated for CD or UC, a universal IUS score for IBD that can be applied in both clinical and research settings is still needed. Our study demonstrated that the IBUS‐SAS has the potential to serve as a universal IUS score in IBD. In conclusion, we believe that the IBUS‐SAS holds great promise for accurately predicting mucosal inflammation in patients with UC and for harmonizing and simplifying IUS in clinical practice and in research settings. Although further confirmation in prospective multi‐centre trials is required, our findings suggest that the IBUS‐SAS could be a valuable tool for clinical decision‐making and as an additional diagnostic marker and endpoint in future clinical trials.

## Ethics Statement

Approval by the ethics committee of the Friedrich‐Alexander‐Universität Erlangen‐Nürnberg (registration number 22‐389‐B).

## Consent

All patients gave informed consent for participation.

## Conflicts of Interest

The authors declare no conflicts of interest.

## Data Availability

The data that support the findings of this study are available on request from the corresponding author. The data are not publicly available due to privacy or ethical restrictions.
